# Triple Iron Chelation in Transfusion Dependent Thalassemia: A Case Report

**DOI:** 10.3390/jcm15082993

**Published:** 2026-04-15

**Authors:** Linet Njue, Emmanuel Häfliger, Alicia Rovó

**Affiliations:** Department of Hematology and Central Hematology Laboratory, Inselspital, Bern University Hospital, University of Bern, 3010 Bern, Switzerland; emmanuel.haefliger@insel.ch (E.H.); alicia.rovo@insel.ch (A.R.)

**Keywords:** beta-thalassemia, transfusion, iron overload, iron chelation

## Abstract

**Background**: Iron overload and its associated complications are major concerns in patients with transfusion-dependent β-thalassaemia (TDT). Iron chelation is an important part of TDT therapy with monotherapy or dual iron chelation being the most commonly used strategies. Evidence regarding the efficacy and safety of triple iron chelation therapy remains limited. **Case presentation**: We present the case of a 21-year-old immigrant from the Middle East with TDT and a history of irregular transfusion management without chelation therapy, leading to clinically significant iron overload. She was successfully treated with the combination of deferoxamine, deferasirox and deferiprone over a course of 8 years. Triple chelation therapy led to sustained reductions in serum ferritin levels and improvement in hepatic and cardiac iron burden on follow-up MRI, with good tolerability. **Conclusions**: This case highlights the potential role of triple iron chelation therapy as a therapeutic strategy in TDT patients with severe iron overload. Further studies are needed to establish optimal dosing, eligible patients and long-term safety.

## 1. Introduction

β-thalassemia is an inherited blood disorder characterized by impaired β-globin synthesis, leading to ineffective erythropoiesis and chronic hemolytic anemia [1]. Patients with transfusion-dependent β-thalassemia (TDT) need lifelong red blood cell transfusions for survival, which consequently results in iron overload. In the absence of proper chelation, iron accumulation inevitably causes progressive complications to organs, especially the heart, liver and endocrine system, which greatly contributes to the morbidity and mortality of these patients [1,2]. Serum ferritin is a commonly used biomarker of iron burden. Additionally, advances in magnetic resonance imaging (MRI) now enable non-invasive direct evaluation of iron concentration in organs, enabling more individualized management [1,2]. MRI techniques evaluate myocardial iron using T2*-weighted imaging and assess liver iron concentration using T2- or T2*-weighted imaging, where T2 and T2* denote relaxation times [2]. Liver iron concentration (LIC) is widely regarded as the best surrogate marker of total body iron stores [2,3]. Table 1 below shows the standard interpretation guidelines for MRI iron assessment.

Iron chelation therapy is the cornerstone of prevention and treatment of iron overload in β-thalassemia. Iron chelation is generally recommended for patients who have received approximately 10 to 20 red blood cell units or have a serum ferritin level above 1000 ng/mL [2,6]. Chelating agents in clinical use include deferoxamine, deferiprone, and deferasirox; each differing in modes of administration, safety characteristics and mode of action. The combination of two iron chelators is used widely in TDT and has been shown to be effective in heavily iron-overloaded patients especially in patients with significant cardiac iron deposition [2,7]. However, experience and published data with triple iron chelation therapy remains limited.

In this report, we describe a TDT patient with severe life-threatening iron overload who was successfully treated with triple iron chelation therapy. This case highlights the clinical rationale, efficacy, and tolerability of an intensified chelation approach in a patient with refractory iron burden and contributes to the limited literature on triple chelation strategies. This report focuses on the patient’s iron chelation therapy. Although appropriate management of her additional comorbidities was an integral component of her treatment, these aspects will not be discussed further in this case report.

## 2. Case Presentation

We present the case of a female TDT patient of Syrian origin, who transitioned to the adult hematology clinic in 2019 at the age of 16 years. The TDT was diagnosed in early childhood, and she had received transfusions irregularly at multiple hospitals while her family was in transit after fleeing their country of origin. A splenectomy had been performed at the age of 11. She had never received any iron chelation before presentation at our hospital. At the time of initial presentation, she was in a reduced clinical state, wheelchair dependent due to severe osteoporosis with numerous pathological fractures and suffered from multiple other comorbidities secondary to iron overload including liver cirrhosis, cardiomyopathy and endocrine and metabolic abnormalities such as diabetes mellitus typ 2, hypothyrodism, hypogonadism and growth impairment.

Her body weight at presentation was 30 kg, at a height of 120 cm. The patient appeared pale and mildly jaundiced. She also demonstrated frontal bossing and maxillary prominence. Abdominal examination revealed a massively distended abdomen with positive shifting dullness suggestive of ascites. Bilateral pedal edema was present. On musculoskeletal examination, there was localized tenderness over the left hip joint on palpation, with reduced range of motion secondary to pain and visible deformity of the affected extremity.

The laboratory results of the patient at initial presentation and currently are summarized in Table 2 below.

Initial MRI examination revealed severe iron overload in the liver and heart with significantly impaired heart function. Her liver iron concentration was 5.06 ms at presentation (normal threshold > 6.3 ms) while her cardiac iron loading was also abnormal at 4.53 ms (normal threshold > 20 ms). See Table 1 above for interpretation of MRI iron assessment.

**Figure 1:**
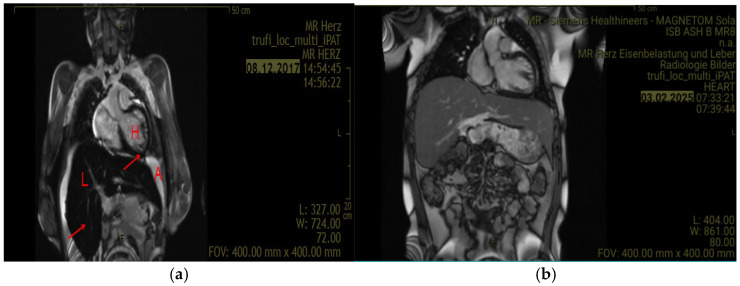
Coronal reconstructed T2*-weighted MRI images demonstrating myocardial and hepatic iron overload before and after iron chelation therapy. (**a**) The left panel shows diffuse hypointense signal (dark areas) in the liver and myocardium, consistent with iron deposition. (**b**) The right panel shows a follow-up MRI performed 7 years later, demonstrating improvement in iron burden (less hypodense signal) following iron chelation therapy. Abbreviations; H = heart, L = liver, Arrow = iron deposits (hypointense (dark) signals), A = ascites.

Figure 1 below illustrates the patient’s first MRI performed at our hospital with multi-echo sequences for T2* quantification and illustrates diffuse hypointense signal of the myocardium and liver on T2*-weighted images, consistent with iron deposition. The figure on the right shows a decrease in the iron deposition (less hypodense) after years of iron chelation.

Regular transfusions were continued at 3-week intervals. Due to the extreme serum ferritin levels and evidence of hepatic and cardiac iron deposition on magnetic resonance imaging, iron chelation therapy with a combination of deferiprone at an initial dose of 2250 mg per day (75 mg/kg/day) in three divided doses and deferasirox at a dose of 540 mg per day (18 mg/kg/day) once daily was initiated in the pediatric clinic when the patient first presented in December 2017. This dual iron chelation strategy was continued until the patient transitioned to the adult clinic 2 years later. Although a moderate reduction in serum ferritin was observed over time, follow-up MRI in June 2018 showed persistent severe iron accumulation in both the liver and heart. In view of the suboptimal response as well as the inadequately controlled multi-organ manifestations described above; a third iron chelator was initiated after transition to the adult clinic to try better control the clinical manifestations. Deferoxamine, at a dose of 1000 mg intravenously after each transfusion, administered at an infusion rate of 15 mg/kg/h, was added to the existing regimen in August 2019. Standard deferoxamine therapy via port was not feasible in this patient due to sociocultural reasons and poor adherence to therapy. This triple iron chelation regimen was continued under close clinical and biochemical monitoring, especially of her renal and hepatic function as well as her blood count.

Common side effects of deferiprone—including gastrointestinal symptoms, transient elevations in liver enzymes, arthropathy, neutropenia, and agranulocytosis, which can sometimes necessitate discontinuation—were not observed in this patient. Notably, her liver enzymes which were elevated prior to starting therapy (likely due to liver cirrhosis), improved after the initiation of iron chelation as is shown in Table 2. Common side effects of deferasirox, such as gastrointestinal disturbances and increased serum creatinine, were also not reported, and her kidney function has remained normal over the years. Since deferoxamine was started in August 2019, the patient has been closely monitored, with no evidence of hypersensitivity reactions, retinopathy, or auditory toxicity. Her pure-tone audiogram has remained normal. In summary, she has not complained of any side effects of this treatment to date.

Although episodes of poor adherence have occurred, treatment interruptions due to medical reasons or side effects have not been necessary. Currently, this triple iron chelation therapy is ongoing. Regular transfusions with a goal of a pretransfusion hemoglobin value of >95 g/L are also ongoing.

**Figure 2:**
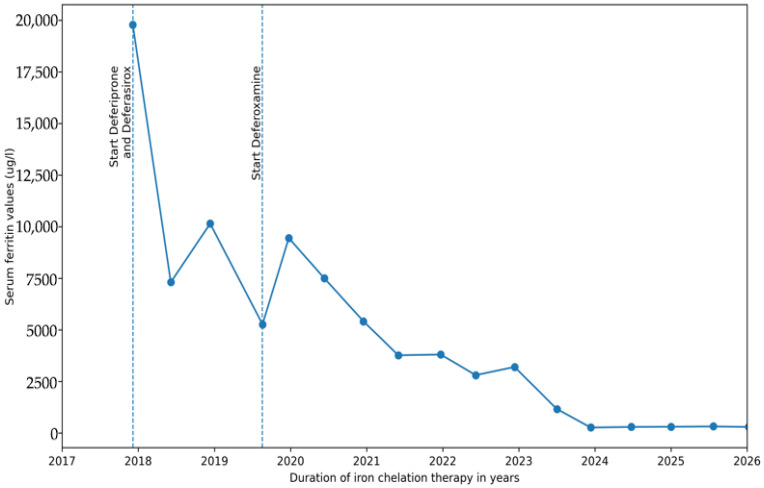
Progressive improvement in serum ferritin levels over time during triple iron chelation therapy. The graph illustrates the longitudinal evolution of serum ferritin concentrations (µg/L, Y-axis) plotted against time in years (X-axis). Each data point represents an individual ferritin measurement obtained at a specific time point throughout the treatment period. A gradual downward trend of ferritin values is observed, reflecting improved control of systemic iron overload over time. The treatment chronology is also illustrated by the dotted vertical lines showing when each drug was initiated; currently triple iron chelation is ongoing.

In her latest follow-up MRI assessment from 02/2025, no iron overload of the liver was documented (T2* 18.45 ms, 1.73 mg/g/dw). Cardiac iron, which typically clears slower than liver iron, was still present though with gradual improvement over time in comparison to previous results (T2* 6.54 ms, 4.89 mg/g/dw). Her serum ferritin levels have dropped from 19,790 µg/L (transferrin saturation 124%) to 291 µg/L (transferrin saturation 84%) over 8 years. Table 3 below presents the MRI results over time, illustrating the decrease in both cardiac and liver iron levels during the course of iron chelation therapy. Figure 2 below illustrates the progressive improvement in serum ferritin levels from the initiation of iron chelation therapy in 2017 to the present as well as the treatment chronology.

## 3. Discussion

Complications arising from iron overload remain significant contributors to morbidity and mortality in patients with TDT. While monotherapy and dual chelation regimens are effective in most patients, some patients with severe or refractory iron overload, particularly involving the liver and heart, may require more intensive therapy. This case describes the successful use of triple iron chelation therapy in a patient with extreme iron burden with a long follow-up period.

To date, only one published study has evaluated triple iron chelation therapy in TDT patients [8]. In this randomized trial, 15 patients were assigned to triple therapy (deferoxamine, deferasirox, and deferiprone) and eight to dual therapy (deferoxamine and deferasirox). Triple therapy was associated with greater reductions in serum ferritin and myocardial iron compared with dual therapy. The study however had a short follow-up period of 6 months.

The use of dual iron chelation therapy has on the other hand been widely studied in TDT [2,7]. The combination of deferoxamine and deferiprone is the most extensively studied and well-established regimen, demonstrating significant reductions in myocardial and hepatic iron accumulation, as well as serum ferritin levels [9]. The combination of deferasirox and deferiprone is particularly attractive from a patient adherence perspective, as both agents are administered orally. Clinical studies have shown this regimen to be an effective and well-tolerated strategy for managing severe iron overload in TDT [10]. Similarly, the combination of deferasirox and deferoxamine has been shown to reduce serum ferritin as well as myocardial and liver iron concentrations [11].

The combination of deferoxamine, deferiprone, and deferasirox may enhance total body iron excretion based on their complementary mechanisms of action. Deferoxamine is effective in mobilizing hepatic iron, deferiprone has great efficacy in removing cardiac iron, with a concordant positive effect on heart function and deferasirox provides sustained systemic chelation [2,7,12]. The rationale for triple therapy lies in the additive and synergistic effects of the three agents, enabling more effective targeting of iron pools in different compartments and potentially improving organ-specific outcomes.

It has to be noted that the standard approach of deferoxamine dosing using subcutaneous infusion via portable pump over hours was not feasible in the presented patient due to sociocultural perspectives and poor compliance to therapy. We therefore opted for an alternative therapy regime as discussed above. As shown in Figure 2 and Table 3, a notable decrease in the patient’s ferritin levels, as well as in cardiac and hepatic iron overload, was observed after the third chelator was introduced. While the expected effect of this intervention alone might be modest, in this case we observed a clear clinical benefit when used in combination with deferasirox and deferiprone. We aim to show that alternative strategies could be considered in select patients, as has been previously shown [13], especially in those with poor adherence to the demanding parenteral use.

In the presented case, triple chelation was initiated following inadequate response to 24 months of optimized dual therapy, as evidenced by persistent markedly elevated serum ferritin levels and significant liver and cardiac iron burden on magnetic resonance imaging. A subsequent improvement in laboratory, clinical and radiological markers of iron overload over time was seen and maintained over several years.

We also show that MRI plays a crucial role in identifying the extent and distribution of iron deposition, guiding appropriate therapeutic decisions and highlighting the value of this investigation in the management of thalassemia-related iron overload. It must also be noted that the appropriate management of the patients’ comorbidities especially cardiac treatment and endocrine dysfunction were an integral part of her clinical care. The patient is no longer wheelchair bound and there is a great improvement in her bone density, as has been shown with combined iron chelation therapy in the literature [14]. Subsequent management, including puberty induction, estrogen supplementation, and calcium and vitamin D supplementation have resulted in improved bone mineralization. The most recent bone densitometry performed in 2024 demonstrated normal bone mass. The patient is currently maintained on calcium 1000 mg and vitamin D_3_ 800 IU daily.

Safety is understandably a major concern with intensified chelation strategies as each agent is associated with distinct adverse effects, including nephrotoxicity, gastrointestinal side effects (deferasirox) [12,15], agranulocytosis (deferiprone) [16], as well as infusion-related complications, ocular and auditory adverse effects (deferoxamine) [2]. In this patient, triple therapy was well tolerated under close clinical and laboratory monitoring, with no serious adverse events observed, highlighting the importance of individualized dosing and vigilant surveillance. Although combination iron chelation therapy is currently not licensed in most regulatory labels, several studies in TDT patients have demonstrated favorable efficacy and safety outcomes [2,7]. Access to these regimens often requires prior authorization from insurance providers as off-label therapy. This case underscores the feasibility and effectiveness of such approaches in clinical practice with the hope of having these combinations licensed in the near future.

Other potential therapeutic strategies that may be considered in severe or refractory iron burden are calcium channel blockers. Since non-transferrin-bound iron can enter cardiomyocytes through L-type calcium channels, calcium-channel blockers like amlodipine can reduce cardiac iron accumulation in transfusion-related iron overload. The use of amlodipine in conjunction with standard chelation therapy has been reported to show significant reduction in serum ferritin levels in TDT patients [17,18].

## 4. Conclusions

In summary, this case underscores the potential role of triple iron chelation therapy in TDT patients with severe, refractory iron overload. While dual chelation remains the standard for many patients, emerging evidence suggests that triple therapy may offer additional benefit in reducing both hepatic and cardiac iron in those not achieving targets with conventional regimens. Careful monitoring and individualized therapy remain essential, and further studies are needed to establish standardized indications, optimal dosing, patient selection, and long-term safety.

## Figures and Tables

**Table 1:** Reference values for quantitative T2 MRI in the assessment of iron overload [4,5].

Myocardial Loading	Myocardial T2*	Hepatic Loading	Hepatic T2*	Dry Weight
None	>20 ms	None	>16 ms	<2 mg/g
Mild	>14–20 ms	Mild	6.4–16 ms	>2–5 mg/g
Moderate	10–14 ms	Moderate	3.2–6.4 ms	5–10 mg/g
Severe	<10 ms	Severe	<3.2	>10 mg/g

Abbreviations: ms: milliseconds, mg/g: milligrams of iron per gram of dry tissue weight.

**Table 2:** Laboratory evolution under triple chelation therapy.

	Laboratory Values at Baseline (8 December 2017)	Laboratory Values After Triple Chelation Therapy (8 January 2026)
Hemoglobin (g/L)	56	88
Hematocrit (%)	15	26
MCV (fL)	74	91
MCH (pg)	27	30
Thrombocytes (G/L)	239	736
Leucocytes (G/L)	23	11.7
Ferritin (µg/L)	19,790	291
Transferrin (g/L)	1.15	1.87
Transferrin saturation (%)	124	84
Glycated hemoglobin (HbA1c) (%)	9.9	8.2
Creatinine (µmol/L)	18	46
Bilirubin (µmol/L)	26	24.6
LDH (U/L)	6341	256
CRP (mg/L)	9	3
ASAT (U/L)	116	21
ALAT (U/L)	68	16
Alkaline phosphatase (U/L)	160	53
Gamma-Glutamyltransferase (U/L)	50	94

Abbreviation: MCV: mean corpuscular volume; MCH: mean content of hemoglobin; LDH: lactate dehydrogenase; CRP: C-reactive protein; ASAT: aspartate aminotransferase; ALAT: alanine aminotransferase.

**Table 3:** MRI results over time.

Date of Examination	Myocardial T2* (ms) /Clinical Category	Liver T2* (ms) /Clinical Category
8 December 2017	4.53/severe	5.06/moderate
21 June 2018	3.3/severe	6.9/mild
27 September 2021	5.3/severe	2.6/moderate
3 February 2025	6.54/severe (improving but still <10 ms)	18.45/normal

Abbreviations: ms: millisecond.

## Data Availability

All data generated for this manuscript is included in the published article. Further inquiries can be directed to the corresponding author.
